# Arthropod but Not Bird Predation in Ethiopian Homegardens Is Higher in Tree-Poor than in Tree-Rich Landscapes

**DOI:** 10.1371/journal.pone.0126639

**Published:** 2015-05-11

**Authors:** Debissa Lemessa, Peter A. Hambäck, Kristoffer Hylander

**Affiliations:** 1 Leuphana University Luneburg, Scharnhorststr 1, Germany; 2 Department of Ecology, Environment and Plant Sciences, Stockholm University, Stockholm, Sweden; Hungarian Academy of Sciences, HUNGARY

## Abstract

Bird and arthropod predation is often associated with natural pest control in agricultural landscapes, but the rates of predation may vary with the amount of tree cover or other environmental factors. We examined bird and arthropod predation in three tree-rich and three tree-poor landscapes across southwestern Ethiopia. Within each landscape we selected three tree-rich and three tree-poor homegardens in which we recorded the number of tree species and tree stems within 100 × 100 m surrounding the central house. To estimate predation rates, we attached plasticine caterpillars on leaves of two coffee and two avocado shrubs in each homegarden, and recorded the number of attacked caterpillars for 7–9 consecutive weeks. The overall mean daily predation rate was 1.45% for birds and 1.60% for arthropods. The rates of arthropod predation varied among landscapes and were higher in tree-poor landscapes. There was no such difference for birds. Within landscapes, predation rates from birds and arthropods did not vary between tree-rich and tree-poor homegardens in either tree-rich or tree-poor landscapes. The most surprising result was the lack of response by birds to tree cover at either spatial scale. Our results suggest that in tree-poor landscapes there are still enough non-crop habitats to support predatory arthropods and birds to deliver strong top-down effect on crop pests.

## Introduction

Top-down control of crop pests is an important ecological service in agroecosystems, but this service may be affected by variation in abundance and distribution of natural enemies across spatial scales [1–4]. Among the factors that influence the abundance and distribution of natural enemies in agroecosystems are dispersal abilities of these organisms [5,6] and habitat heterogeneity [7–9]. Habitats differ in their potential of resource provisioning to different organisms [10–13] and thereby affect the diversity of crop pest regulators [14,15]. For example, the variation in habitat suitability for foraging, nesting and breeding mediates the abundance and diversity of birds [16–18] and such variations may in turn influence the top-down effects of birds on crop pests [19–21]. Moreover, isolation or lack of connectivity among habitats may limit the movement of birds in agricultural landscapes and may dampen their top-down effect on crop pests [19,22].

Studies comparing complex and simple habitats have noted that complex habitats attract more insectivorous birds due to the availability of habitats with high quality resources for nesting and foraging [23–25], and also for a favorable microclimate [20,26,27]. Several studies have found higher bird predation in habitats with higher tree cover [28–31]. In contrast, other studies have shown less effect of tree structure on bird predation [32–34] or higher bird predation in agroforestry systems (i.e., simplified habitats) than in natural forests [8,20,35]. Similarly, many studies have shown that habitat complexity benefits arthropod natural enemies and thereby pest control in agricultural landscapes [36,37]. One mechanism underlying this pattern is that complex habitats provide better refuges or reduce contact rates between competing predators so that intraguild predation is reduced [38]. Other studies have in contrast shown weaker top-down effects of predatory arthropods on crop pests in complex landscapes due to higher negative interaction from top predators (e.g. birds feeding on predatory flying insects) compared to in simple landscapes [21]. Hence, the management initiatives to enhance the natural pest control needs a better understanding of how these ecosystem services are mediated by the variation in vegetation structure across agricultural landscapes [39].

In many tropical homegardens, farmers grow a diversity of perennial and annual crops (vegetables, fruit trees and spices) integrated with medicinal, ornamental, fodder and fuel wood plants [40–43]. Tropical homegardens vary considerably in vegetational structure [44,45], but the effect of vegetation cover gradients on natural pest control services has nevertheless received little attention in tropical regions. This lack of information is unfortunate as the benefit could be high because insecticides are typically too costly for many farmers in tropical areas to use in their homegardens [46–51]. In southwest Ethiopia, the agricultural landscapes are mosaics of small land-use types which may influence the pest suppression services provided by natural enemies [47,52]. In this study, we hypothesized that: (1) The rates of predation on caterpillars by birds and predatory arthropods are higher in tree-rich homegardens and tree-rich landscapes as these areas provide predators with more refuges and alternative food resources than tree-poor homegardens and tree-poor landscapes, (2) The rates of predation show a larger difference between tree-rich and tree-poor homegardens in tree-poor landscapes since tree-poor homegardens in these landscapes are less connected to buffering habitats than tree-poor homegardens in tree-rich landscapes.

Our overall results showed that the arthropod predation rates were higher in tree-poor than in tree-rich landscapes, but no such difference was found for bird predation rates, which is surprising and contradictory to our hypothesis. In tree-poor landscapes, the arthropod predation rates were also higher than the bird predation rates, while there was no such significant difference in tree-rich landscapes.

## Materials and Methods

### Study areas

The study was conducted in agricultural landscapes of Jimma zone, southwest Ethiopia (7°24^' –^ 8°4' N and 35°58' – 37°14' E). The study landscapes lie within the altitudinal range of 1600−2250 m a.s.l. and their topography varies from gentle to undulating and rugged slopes. The major soil type is Nitosol [53,54]. The areas get high rainfall from May to September and the rainfall per annum varies between 1150 and 2080 mm. The mean monthly temperature varies between 10 and 26°C [54].

The landscapes are comprised of a mixture of agricultural crop fields, fallow lands, grazing lands, homegardens and live fences, remnants of small and large natural forest patches and wetlands. The forest type is moist evergreen afromontane forest [55] and is comprised of tree species such as *Pouteria adolfi-friederici*, *Croton macrostachyus*, *Schefflera abyssinica*, *Allophyllus abyssinicus*, *Cordia africana*, *Millettia ferruginea*, *Sapium ellipticum*, *Albizia* spp., *Prunus africana* and *Olea welwitschii*. The farming communities grow annual crops including teff (*Eragrostis teff*), maize, barley, sorghum, wheat, pulses, finger millet, oil crops and perennial crops including coffee (*Coffea arabica*), khat (*Catha edulis*), ensete (*Ensete ventricosum*) and fruit trees. In most of the landscapes, but mainly at lower altitudes (below 2000 m a.s.l.), farmers grow coffee as semi-natural forest and garden coffee [56]. Khat grows across the whole altitudinal range. Ensete is a perennial herbaceous plant that is commonly called “false banana” due to its morphological similarity with banana. In the area, ensete abundantly grows in homegardens and people use its pseudostem and corm for household consumption.

### Study design

We used satellite images from Google Earth to identify six landscapes with an approximate size of 10 x 10 km and having either high or low forest cover. Three landscapes had > 30% forest cover and were defined as tree-rich and three landscapes had < 10% forest cover and were defined as tree-poor. The minimum and maximum straight line distance between the landscapes were 18 km and 85 km respectively. Within each landscape, we selected three tree-poor homegardens and three tree-rich homegardens based on variation in tree cover within 100 × 100 m surrounding the house besides assessed from the satellite images. From the satellite images we identified tree-poor homegardens with a dominance of open fields and tree-rich homegardens with a high tree cover surrounding the focal house. Tree cover also included perennial crops grown such as coffee, ensete, khat and fruit trees, since these land-use types could not be separated from other trees or shrubs in the satellite images. In total, we selected 36 homegardens in a full factorial design. All landscapes and homegardens were visited with the permission from local administrative persons and homegarden owners and no permits for field data collection were required.

In each homegarden we recorded tree species and number of tree stems (≥ 10 cm dbh). To measure the level of predation (by birds and predatory arthropods) across the six landscapes, we used plasticine caterpillars that should mimic a representative light green-colored caterpillar. We were unable to mimic a specific species as we had poor knowledge on the caterpillars that normally feed on either of our target plants. However, the dominant arthropod and bird predators are assumed to be generalist predators and would attack most green-colored caterpillars.

The use of plasticine caterpillars for estimating predation rates is widely applied in the tropics, it is simple and does not need sophisticated equipment [3,57,58]. The plasticine caterpillars (diameter = 2.5 and length = 10 mm) were produced from non-toxic light green hobby modeling clay (Staedtler, 8420 C10, http://www.pandurohobby.se/Katalog/80-Barn-Junior/8040-Pyssla/804010-Lera/1/049882-Modellera-21g-10-farger) using a garlic squeezer. After selecting two avocado and two coffee shrubs located at different places within the homegarden, we attached 25 plasticine caterpillars on the leaves of each of four shrubs (i.e., 100 plasticine per each homegarden), approximately between 1–1.5 m heights from the ground, using liquiSole glue (Casco, Spackellimen, http://www.hilevel.se/en/casco-glue/liquisole-glue.html). In one landscape we were unable to find avocado shrubs and attached all caterpillars on the leaves of four coffee shrubs. In total, 600 plasticine caterpillars (i.e., 100 in each of six homegardens) were initially placed in each landscape at the start of the study which sums up to a total of 3600 caterpillars. We scored the number of plasticine caterpillars attacked, removed or missing three times per week (either Monday-Wednesday-Friday or Tuesday-Thursday-Saturday) for seven to nine consecutive weeks (26-Aug-2013 to 25-Oct-2013). During each data collection event, all caterpillars that were either attacked or missing were replaced by new ones at the same place. We also replaced non-attacked caterpillars that had faded colors due to the effect of sunlight and rainfall.

We adopted the identification of attack marks for different predator organisms (birds or arthropods) developed by Howe et al. [58] and Tvardikova and Novotny [3]. The predatory arthropods that were observed attacking or roaming around the plasticine caterpillars were ants, wasps and predatory beetles (e.g. fireflies). We did not distinguish the attack marks by different predatory arthropods and as a result our predation data was based on two groups: bird predation and the combined predation by all predatory arthropods. We did not observe any damage from mammals on the caterpillars. Removed or missing plasticine caterpillars were excluded from the data analysis since their status could not be assessed.

### Statistical analyses

For all the statistical analyses we used the statistical program R version 2.15.3 [59]. We first compared the number of tree species and the number of tree stems among homegardens and among landscapes in a linear mixed effects model using *lmer* function in the lme4-package [60]. Before analyses, we transformed the two variables using square root transformation to normalize their distributions. As predictive variables, we used garden type (tree-rich or tree-poor), landscape type (tree-rich or tree-poor) and altitude (standardized) as fixed factors and landscape identity as random factor. We ran this analysis by first including all variables and two-way interactions and then performed a backward selection until only variables significantly contributing to the models remained [61].

The daily predation rates were calculated as the number of attacked plasticine caterpillars divided by the number of exposed minus missed caterpillars, corrected for the time interval (two or three days) and multiplied by 100. First, to test the effect of the time (a repeated measure over seven to nine weeks), the variation in daily predation rates (for birds and arthropods, separately) was analyzed using a linear mixed effects model, with garden type (tree-rich or tree-poor), landscape type (tree-rich or tree-poor) and dates of data collection (from 26 August 2013 to 25 October 2013) as fixed factors, and with landscape identity (six landscapes) and also dates (nested within landscape identity (n = 6)) as random factors. However, since there was no effect of time or dates on the predation rates for both birds and arthropods (lmer, *p* > 0.48, Table A1 and A2 in S1 Tables), we excluded this variable and calculated the mean daily predation rates per garden over the whole period which we used as response variable for the further analysis. Hence, the homegarden was the level of replication for the analyses of the mean daily predation rates. Second, in the linear mixed effects model, we retained garden type and landscape type as fixed factors and landscape identity as random factor. Models were separately fitted for the two bird and arthropod predation response variables.

To further compare predation rates among landscapes, separately for birds and predatory arthropods, we used a linear mixed effects model with the landscape identity (n = 6) as fixed factor and garden type nested under landscape type as random factors. To determine the variation explained due to the random effects of landscapes and homegardens, a variance partitioning analysis was performed for bird and arthropod predation separately. To analyze the difference between bird and arthropod predation rates we used a linear mixed effects model with predator type (bird or arthropod), landscape type, garden type and their two-way interactions as fixed factors and landscape identity as a random factor. The effect of altitude on predation rates was separately analyzed for birds and arthropods using linear mixed effects model with additional variables landscape type (fixed factor) and landscape identity (random factor). Finally, we compared the mean daily predation rates on plasticine caterpillars (by birds and predatory arthropods, separately) between plant types (coffee vs. avocado) using linear mixed effects model with landscape identity as a random factor. This analysis was made for the sub dataset of 5 landscapes where we had predation data on both plant types. The assumptions of normality of residuals and homoscedasticity were inspected from the residual plots of the final models.

## Results

### Variation in tree cover

**Fig 1:**
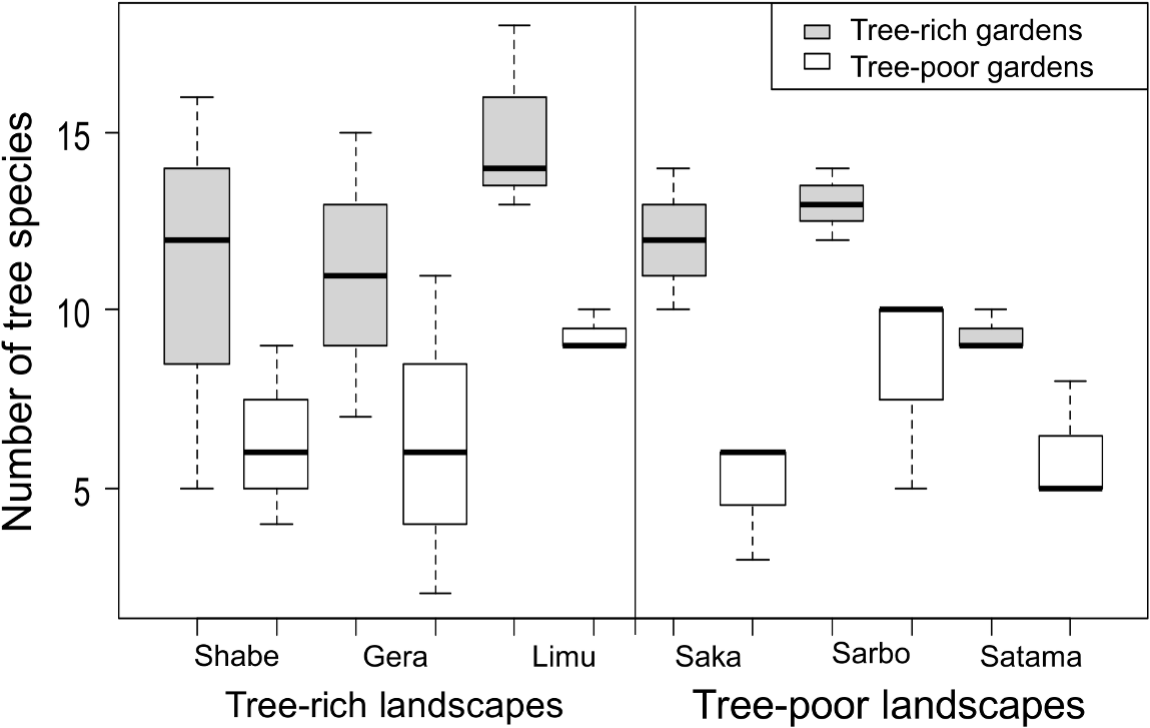
The variation in number of tree species between tree-rich and tree-poor homegardens, across tree-rich and tree-poor landscapes.

The number of tree species and number of tree stems differed between tree-rich and tree-poor homegardens (lmer, *p* < 0.001, Fig 1), but not between tree-rich and tree-poor landscapes (*p* > 0.75), validating our subdivision in garden and landscape types. Altitude had no effect on the variation in either tree cover variable.

### Predation on plasticine caterpillars

Overall, we recorded a total of 5155 attacks on caterpillars (pooled over time), of which 2440 were attacks by birds and 2715 by predatory arthropods. The total number of missed caterpillars was 4176 (but they were replaced). Since it was not possible to identify if the missed caterpillars were attacked or not and if attacked by which predators we excluded them from the analysis.

#### Bird predation

**Fig 2:**
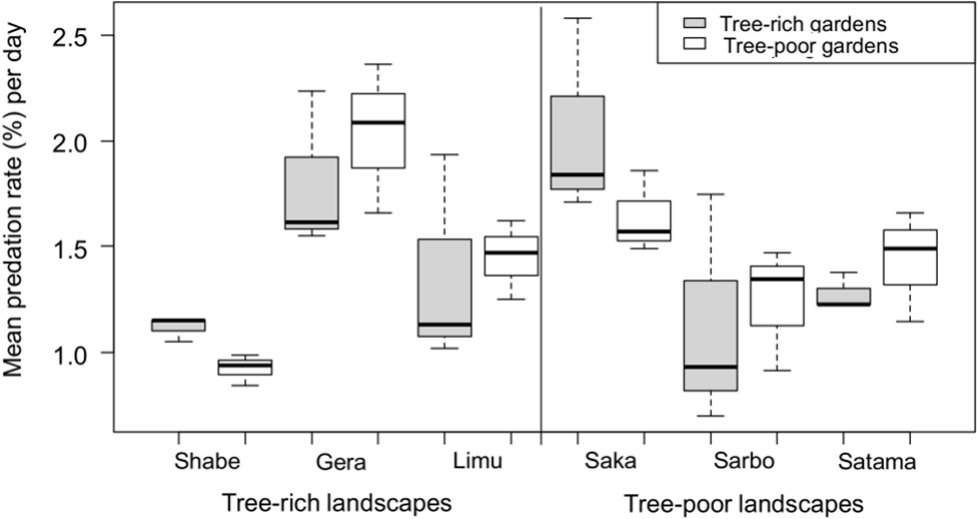
The daily bird predation rates in tree-rich and tree-poor homegardens within tree-rich and tree-poor landscapes.

The mean daily predation rate by birds on the plasticine caterpillars was 1.40% (range: 0.8–2.4%) in tree-rich landscapes and 1.46% (range: 0.7–2.6%) in tree-poor landscapes. The bird predation rates varied among landscapes (lmer, *p* < 0.001, Fig 2), but did not differ systematically between landscape types, homegarden types or by the interaction between landscape and homegarden types (lmer, *p* > 0.69, Table 1).

**Table 1:** Results from linear mixed effects model analyses on bird and arthropod predation rates on plasticine caterpillars.

Models	Factors	DF	χ^2^	p
1. Bird predation	Landscape type	1	0.001	0.97
	Garden type	1	0.0002	0.99
	Landscape type: Garden type	1	0.154	0.69
2. Predatory arthropods	Landscape type	1	8.55	0.003
	Garden type	1	0.071	0.79
	Landscape type: Garden type	1	0.752	0.39

Landscape identity (six landscapes) was included as a random variable.

Bird predation rates were not affected by either altitude (lmer, *p* = 0.37, Table B1 in S1 Tables) or plant species (avocado vs. coffee) (lmer, *p* = 0.54, Table C1 in S1 Tables). In the model, 56% of the variance was accounted for by the random effect of landscape identity while 44% of the variance was due to variation between homegardens (Fig 2).

#### Arthropod predation

**Fig 3:**
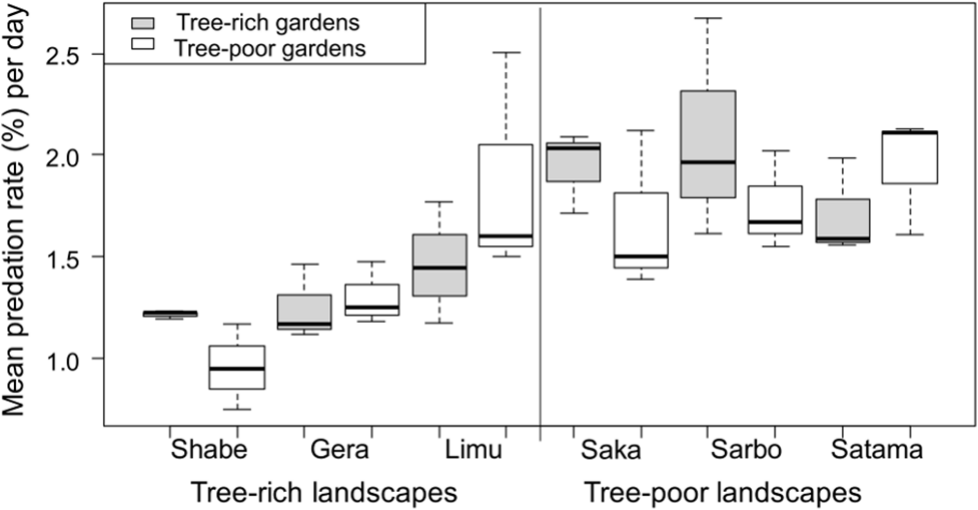
The rates of arthropod predation per day in tree-rich and tree-poor homegardens within tree-rich and tree-poor landscapes.

The mean daily arthropod predation rates on plasticine caterpillars were 1.34% (range: 0.7–2.5%) in tree-rich landscapes and 1.85% (range: 1.4–2.7%) in tree-poor landscapes. The arthropod predation rates varied among landscapes (*p* < 0.001, Fig 3), and were systematically higher in tree-poor than in tree-rich landscapes (lmer, *p* = 0.003, Table 1), but did not vary between tree-rich and tree-poor homegardens (*p* = 0.79, Table 1). Arthropod predation rates were not affected by either altitude (lmer, *p* = 0.27, Table B2 in S1 Tables) or plant species (coffee vs. avocado) (lmer, *p* = 0.53, Table C2 in S1 Tables). In the models of arthropod predation, 52% of the variance was accounted for by the variation among landscapes while 48% of the variance was due to variation among homegardens (Fig 3).

#### Birds vs. arthropod predation

**Fig 4:**
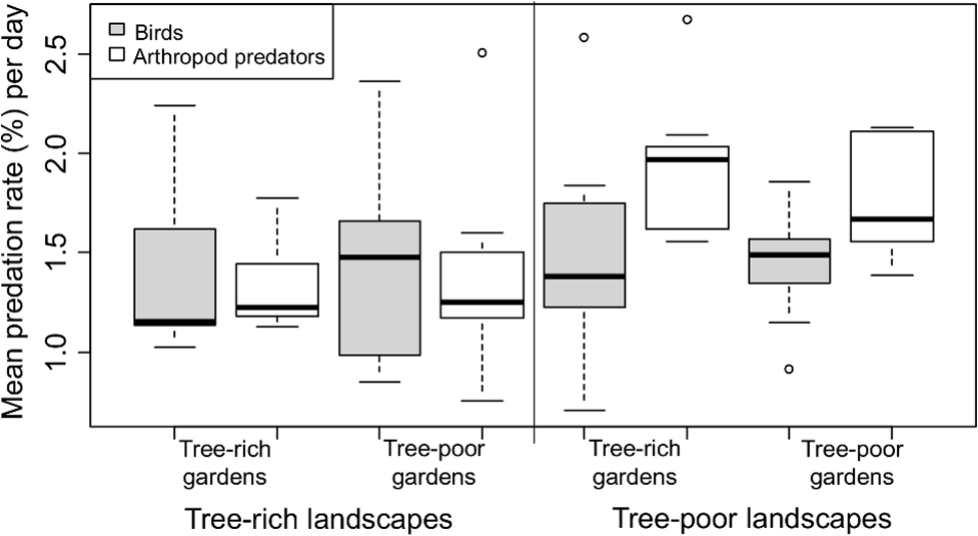
Bird and arthropod predation rates in tree-rich and tree-poor homegardens within tree-rich and tree-poor landscapes.

The overall predation rate was affected by the interaction between landscape type and the type of predator organism (lmer, *p* = 0.004, Table D in S1 Tables). The interaction effect appeared mainly because the predation rates of arthropods on plasticine caterpillars were higher than those of birds in tree-poor landscapes, while there were no differences between these predation rates in tree-rich landscapes (Fig 4).

## Discussion

When comparing predation rates on plasticine caterpillars between tree-rich and tree-poor landscapes in southwest Ethiopia, we show that arthropod predation rates were higher in tree-poor than in tree-rich landscapes. No such difference was found for bird predation rates. The arthropod predation rates were also higher than the bird predation rates in tree-poor landscapes, while the arthropod and bird predation rates were not different in tree-rich landscapes (Fig 4). The effect of homegarden tree cover was limited, as we found no difference in either arthropod or bird predation rates between tree-rich and tree-poor homegardens within either tree-rich or tree-poor landscapes. The mean daily bird predation rate in our area was 1.45%, which is lower than the predation rates (approx. 11%) found in gaps and understory of tropical forests during dry and wet seasons in Panama [62]. However, the daily predation rates that we recorded in homegardens were within the range of daily predation rate recorded in cotton plots (1.1–8.9) in Uganda [58]. The mean daily arthropod predation rate in our area was 1.60%, which is lower than the 3.1% found on plasticine caterpillars in Papua New Guinea from various forest habitats [3].

The effect of vegetation cover on bird predation rates seems to vary among studies. Although several studies have indicated the importance of vegetation cover for bird predation [9,28–31], several studies including ours rather indicated small or no effects of vegetation structure on bird predation [32–34,63]. Some studies have in contrast found higher bird predation in disturbed or in fragmented and simplified habitats than in natural forests, although those habitats still are structurally rather complex [20,29,35]. Hence, the effect of vegetation cover gradient on patterns of bird predation seems variable and further studies should focus on the potential mechanisms underlying this variation. One possible explanation for variation could be differences in the spatial scale of the studies. Many birds have a high mobility and may also be adapted to the habitat disturbance by human land-use [64,65]. It seems that the bird diversity in our study area may be higher in homegardens compared to forests [66,67]. Engelen [67] recorded 58 bird species, which include many insectivorous ones (48%) in tree rich homegardens close to our study sites. Among the insectivore birds that were commonly observed feeding in the understory of homegardens during the dry and wet seasons were Tawny-flanked Prinia (*Prinia subflava)*, Grey-backed Camaroptera (*Camaroptera brachyura*), and Rüppell's Robin-Chat (*Cossypha semirufa*). Within the agricultural landscapes, bird species richness may nevertheless increase with tree cover [68], which may indicate higher predation rates in such settings provided that there is a correlation between species richness and bird predation rates. Unfortunately we have not estimated either species richness or abundances of birds in the experimental homegardens in this study.

There are many findings indicating that habitats with higher vegetation cover enhance the top-down control of predatory arthropods in agricultural landscapes [36–38,69]. Our finding contrasts with those studies and is more consistent with a smaller set of studies showing higher arthropod predation in tree-poor than in tree-rich landscapes [21,70]. Our results are also similar to a study conducted in tropical forests of Papua New Guinea by Tvardikova and Novotny [3] that found higher predation by insect predators (e.g. by ants and wasps) on caterpillars in disturbed or fragmented areas than in continuous forest areas even if these areas could still be more structurally complex than our tree poor homegardens.

In an earlier study, we found that the dominant ground predatory arthropods recorded from pitfall traps in the homegardens of southwest Ethiopia were ants, spiders and beetles [71,34]. The abundance of ants (mostly army ants, *Dorylus* sp.) generally increased with tree cover while the spider abundance (mostly wolf spiders, Lycosidae) had a more mixed response to tree cover. The homegardens with the highest spider abundance were those with a high tree cover either within the homegarden or in the surrounding landscape [71]. Homegardens with a high or low tree cover both within the homegarden and in the surrounding landscape had lower spider abundance. Based on these result, three alternative hypotheses may explain our observation of higher arthropod predation in tree-poor than in tree-rich landscapes. First, the most likely hypothesis is that tree-poor landscapes simply had a higher number of predators, presumably because these habitats also harbor a higher number of prey individuals (attracted by surrounding annual crops). The higher numbers of predatory arthropods in tree-poor landscapes would then cause higher predation rates compared to in tree-rich landscapes. However, spiders are not likely to attack plasticine caterpillars since they most often attack moving prey [72]. Second, the plasticine larvae could also be less attacked in tree-rich landscapes if these areas have a higher density of prey per area (but not per shrub or tree) and that the predators are then less eager to attack the plasticine caterpillars. Third, the community structure of prey could be different between landscapes such that predators in the different landscapes seek different prey. One could speculate that our model caterpillars may represent different prey to the predators in the different settings and that this may explain differences in predation rates. Although it was suggested that the use of dummy caterpillars method is novel, our results highlight the need for further studies comparing real predation rates with predation on caterpillars [58].

The rates of bird and also arthropod predation varied among the landscapes within categories (Figs 2 and 3), suggesting that the landscapes may also vary in some aspects which could be important for predator abundances; but this was not examined with the present study. In conclusion, our results underscore the potential importance of heterogeneous habitats in tree-poor landscapes of tropical agroecosystems in supporting predatory arthropods to the level that they can deliver a strong top-down effect on crop pests [71]. However, we did not find clear evidence for the effect of the tree cover variation on the rate of bird predation.

## Supporting Information

S1 Tables The linear mixed effects model analysis for the daily predation rates. First step analyses with repeated measure using linear mixed effects model to test the effect of data collection time on predation rates of birds (Table A1) and arthropods (Table A2). The linear mixed effects model analysis on the effect of altitude on predation rates of birds (Table B1) and arthropods (Table B2). The linear mixed effects model analysis on the effect of plant type (coffee and avocado, on which plasticine caterpillars were attached) on predation rates of birds (Table C1) and arthropods (Table C2). The linear mixed effects model analysis to test the difference in predation rates between birds and arthropods on plasticine caterpillars (Table D) (PDF).
(PDF)

## References

[1] Pearce S, Zalucki MP. Do predators aggregate in response to pest density in agroecosystems? Assessing within-field spatial patterns. Journal of Appl Ecol. 2005; 43: 128–140.

[2] Tscharntke T, Klein AM, Kruess A, Steffan-Dewenter I, Thies C. Landscape perspectives on agricultural intensification and biodiversity- ecosystem service management. Ecol Lett.2005; 8: 857–874.

[3] Tvardikova K, Novotny V. Predation on exposed and leaf-rolling artificial caterpillars in tropical forests of Papua New Guinea. J Tropic Ecol. 2012; 28: 331–341.

[4] Ferrante M, Lo Cacciato A, Lövei GL. Quantifying predation pressure along an urbanization gradient in Denmark using artificial caterpillars. Eur J Entomol. 2014; 111: 649–654.

[5] Amarasekare P, Nisbet RM. Spatial heterogeneity, source-sink dynamics, and the local coexistence of competing species. Am Nat. 2001; 158: 572–584. 10.1086/323586 18707352

[6] Tscharntke T, Brandl R. Plant-insect interactions in fragmented landscapes. Ann Rev Entomol. 2004; 49: 405–430. 14651470 10.1146/annurev.ento.49.061802.123339

[7] Wilby A, Lan LP, Heong KL, Huyen NPD, Quang NH, Minh NV et al Arthropod diversity and community structure in relation to land use in the Mekong Delta, Vietnam. Ecosyst. 2006; 9: 538–549.

[8] Zaviezo T, Grez AA, Estades CF, Pérez A. Effects of habitat loss, habitat fragmentation, and isolation on the density, species richness, and distribution of ladybeetles in manipulated alfalfa landscapes. Ecol Entomol. 2006; 31: 646–656.

[9] Bereczki K, Ódor P, Csóka G, Mag Z, Báldi A. Effects of forest heterogeneity on the efficiency of caterpillar control service provided by birds in temperate oak forests. For Ecol Manage. 2014; 327: 96–105.

[10] Roland J, Taylor PD. Insect parasitoid species respond to forest structure at different spatial scales. Nature. 1997; 386: 710–713.

[11] Rand TA, Tylianakis JM, Tscharntke T. Spillover edge effects: the dispersal of agriculturally subsidized insect natural enemies into adjacent natural habitats. Ecol let. 2006; 9: 603–614. 16643305 10.1111/j.1461-0248.2006.00911.x

[12] With KA, Pavuk DM, Worchuck JL, Oates RK, Fisher JL. Threshold effects of landscape structure on biological control in agroecosystems. Ecol Appl. 2002; 12: 52–65.

[13] O’Rourke M, Rienzo-Stack K, Power AG. A multi-scale, landscape approach to predicting insect populations in agroecosystems. Ecol Appl. 2011; 21: 1782–1791. 21830718 10.1890/10-0241.1

[14] Tscharntke T, Şekercioğlu C, Dietsch TV, Sodhi NS, Hoehn P, Tylianakis JM. Landscape constraints on functional diversity of birds and insects in tropical agroecosystems. Ecol. 2008; 89: 944–951. 18481519 10.1890/07-0455.1

[15] Thies C, Haenke S, Scherber C, Bengtsson J, Bommarco R, Clement LW et al The relationship between agricultural intensification and biological control: experimental tests across Europe. Ecol Appl. 2011; 21: 2187–2196. 21939053 10.1890/10-0929.1

[16] Jobin B, Choinièreb L, Bélangera L. Bird use of three types of field margins in relation to intensive agriculture in Québec, Canada. Agric Ecosyst Environ. 2001; 84:131–143.

[17] Batáry P, Matthiesen T, Tscharntke T. Landscape-moderated importance of hedges in conserving farmland bird diversity of organic vs. conventional croplands and grasslands. Biol Conserv. 2010; 143: 2020–2027.

[18] Watson JEM, Whittaker RJ, Freudenberger D. Bird community responses to habitat fragmentation: how consistent are they across landscapes? J Biogeogr. 2005; 32: 1353–1370.

[19] Şekercioğlu ÇH, Ehrlich PR, Daily GC, Aygen D, Goehring D, Sandi RF. Disappearance of insectivorous birds from tropical forest fragments. Proc Natl Acad Sci USA. 2002; 99: 263–267. 11782549 10.1073/pnas.012616199 PMC117549

[20] Ruiz-Guerra B, Renton K, Dirzo R. Consequences of fragmentation of tropical moist forest for birds and their role in predation of herbivorous insects. Biotropica. 2012; 44: 228–236.

[21] Martin EA, Reineking B, Seo B, Steffan-Dewenter I. Natural enemy interactions constrain pest control in complex agricultural landscapes. Proc Natl Acad Sci. 2013; 110: 5534–5539. 10.1073/pnas.1215725110 23513216 PMC3619341

[22] Patten MA, Bolger DT. Variation in top-down control of avian reproductive success across a fragmentation gradient. Oikos. 2003; 3: 479–488.

[23] Johnson RJ, Beck MM. Influence of shelterbelts on wildlife management and biology. Agric Ecosyst Environ. 1988; 22: 301–335.

[24] Wilson JD, Evans J, Browne SJ, King JR. Territory distribution and breeding success of Skylarks *Alauda arvensis* on organic and intensive farmland in southern England. J Appl Ecol. 1997; 34: 1462–1478.

[25] Gunnarsson B, Heyman E, Vowles T. Bird predation effects on bush canopy arthropods in suburban forests. Forest Ecol Manag. 2009; 257: 619–627.

[26] Gillis H, Gauffre B, Huot R, Bretagnolle V. Vegetation height and egg coloration differentially affect predation rate and overheating risk: an experimental test mimicking a ground-nesting bird. Canad J Zool. 2012; 90: 694–703.

[27] De La Vega X, Grez AA, Simonetti JA. Is top-down control by predators driving insect abundance and herbivory rates in fragmented forests? Austr Ecol. 2012; 37: 836–844.

[28] Perfecto I, Vandermeer JH, Bautista GL, Nuñez GI, Greenberg R, Bichier P et al Greater predation in shaded coffee farms: the role of resident neotropical birds. Ecol. 2004; 85: 2677–2681.

[29] Van Bael SA, Philpott SM, Greenberg R, Bichier P, Barber NA, Mooney KA et al Birds as predators in tropical agroforestry systems. Ecol. 2008; 89: 928–934. 10.1890/06-1976.1 18481517

[30] Johnson MD, Levy NJ, Kellermann JL, Robinson DE. Effects of shade and bird exclusion on arthropods and leaf damage on coffee farms in Jamaica’s blue mountains. Agroforest Syst. 2009; 76: 139–148.

[31] Johnson MD, Kellermann JL, Stercho AM. Pest reduction services by birds in shade and sun coffee in Jamaica. Anim Conserv. 2010; 13: 140–147.

[32] Gunnarsson B. Bird predation and vegetation structure affecting spruce-living arthropods in a temperate forest. J Anim Ecol. 1996; 65: 389–397.

[33] Philpott SM, Soong O, Lowenstein JH, Pulido AL, Lopez DT, Flynn DFB et al Functional richness and ecosystem services: bird predation on arthropods in tropical agroecosystems. Ecol Appl. 2009; 19: 1858–1867. 19831075 10.1890/08-1928.1

[34] Van Bael SA, Brawn JD. The direct and indirect effects of insectivory by birds in two contrasting Neotropical forests. Oecologia. 2005; 145: 658–668. 16315345 10.1007/s00442-005-0134-0

[35] Barbaro L, Brockerhoff EG, Giffard B, van Halder I. Edge and area effects on avian assemblages and insectivory in fragmented native forests. Land Ecol. 2012; 27: 1451–1463.

[36] Bianchi FJJA, Booij CJH, Tscharntke T. Sustainable pest regulation in agricultural landscapes: a review on landscape composition, biodiversity and natural pest control. Proc Roy Soc B. 2006; 273: 1715–1727. 16790403 10.1098/rspb.2006.3530 PMC1634792

[37] Rusch A, Bommarco R, Jonsson M, Smith HG, Ekbom B. Flow and stability of natural pest control services depend on complexity and crop rotation at the landscape scale. J Appl Ecol. 2013; 50: 345–354.

[38] Janssen A, Sabelis MW, Magalhães S, Montserrat M, van der Hammen T. Habitat structure affects intraguild predation. Ecol. 2007; 88: 2713–2719. 18051638 10.1890/06-1408.1

[39] Bianchi FJJA, Schellhorn NA, Cunningham SA (2013) Habitat functionality for the ecosystem service of pest control: reproduction and feeding sites of pests and natural enemies. Agric Forest Entomol 15: 12–23.

[40] Kumar BM, Nair PKR. The enigma of tropical homegardens. Agroforest Syst. 2004; 61: 135–152.

[41] Nair PKR. Do tropical homegardens elude science, or is it the other way around? Agroforest Syst. 2001; 53: 239–245.

[42] Soemarwoto O. Homegardens: a traditional agroforestry system with a promising future In: Steppler HA and Nair PKR, editors. Agroforestry: A Decade of Development, ICRAF, Nairobi, Kenya; 1987 pp. 157–170.

[43] Singh GB. Agroforestry in the Indian subcontinent, past, present and future In: Steppler HA and Nair PKR, editors. Agroforestry: A Decade of Development: Nairobi, Kenya; 1987 pp. 117–140.

[44] Albuquerque UP, Andrade LHC, Caballero J. Structure and floristics of homegardens in Northeastern Brazil. J Arid Environ. 2005; 62: 491–506.

[45] Abebe T, Wiersum K F, Bongers F. Spatial and temporal variation in crop diversity in agroforestry homegardens of southern Ethiopia. Agroforest Syst. 2010; 78: 309–322.

[46] Girma H, Rao MR, Sithanantham S. Insect pests and beneficial arthropod populations under different hedgerow intercropping systems in semiarid Kenya. Agroforest Syst. 2000; 50: 279–292.

[47] Abate T., van Huis A, Ampofo J K . Pest management strategies in traditional agriculture: an African perspective. Ann Rev Entomol. 2000; 45: 631–659. 10761592 10.1146/annurev.ento.45.1.631

[48] Banwo OO, Adamu RS. Insect pest management in African agriculture: challenges in the current millennium. Archives Phytopathol Plant Protect. 2003; 36:59–68.

[49] Otieno M, Woodcock BA, Wilby A, Vogiatzakis IN, Mauchline AL, Gikungu MW et al Local management and landscape drivers of pollination and biological control services in a Kenyan agro-ecosystem. Biol Conserv. 2011; 144: 2424–2431.

[50] Howe AG, Nachman G, Lövei GL. Predation pressure in Ugandan cotton fields measured by a sentinel prey method. Entomol Exp Appl. 2015; 154: 161–170.

[51] MacFadyen S, Davies AP, Zalucki MP. Assessing the impact of arthropod natural enemies on crop pests at the field scale. Insect Sci. 2015; 22:20–34. 10.1111/1744-7917.12174 25219624

[52] Jones GA, Sieving KE, Jacobson SK. Avian diversity and functional insectivory on North-Central Florida farmlands. Conserv Biol. 2005; 19: 1234–1245.

[53] Dubale P. Soil and water resources and degradation factors affecting productivity in Ethiopian highland agroecosystems. Northeast Afric Stud. 2001; 8: 27–51.

[54] Nigussie A, Kissi E. Physicochemical Characterization of Nitisol in Southwestern Ethiopia and Its Fertilizer Recommendation Using NuMaSS. Glo Adv Res J Agric Sci. 2012; 1:66–73.

[55] Friis I, Demissew S, van Breugel P. Atlas of the potential vegetation of Ethiopia. Det Kongelige Danske Videnskabernes Selska, Specialtrykkeriet Viborg a-s, Copenhagen, Denmark; 2010.

[56] Teketay D. History, botany and ecological requirements of coffee. Walia. 1999; 20:28–50.

[57] Koh LP, Menge DNL. Rapid assessment of lepidopteran predation rates in Neotropical forest fragments. Biotropica. 2006; 38: 132–134.

[58] Howe A, Lövei GL, Nachman G. Dummy caterpillars as a simple method to assess predation rates on invertebrates in a tropical agroecosystem. Entomol Experim et Appl. 2009; 131: 325–329.

[59] R Development Core Team R: A language and environment for statistical computing R foundation for statistical computing, Vienna, Austria ISBN 3-900051-07-0; 2013 Available: URL http://www.R-project.org/.

[60] Bates D, Maechler M, Bolker B. lme4: Linear mixed-effects models using S4 classes. R package version 0.999375–42. 2013. Available: http://CRAN.R-project.org/package=lme4.

[61] Gotelli NJ, Ellison AM. A Primer of ecological statistics, Sinauer Associates, inc Publishers Sunderland, Massachusetts; 2004.

[62] Richards LA, Coley PD. Seasonal and habitat differences affect the impact of food and predation on herbivores: a comparison between gaps and understory of a tropical forest. Oikos. 2007; 116: 31–40.

[63] Greenberg R, Bichier P, Angon AC, MacVean C, Perez R, Cano E. The impact of avian insectivory on arthropods and leaf damage in some Guatemalan coffee plantations. Ecol. 2000; 81: 1750–1755.

[64] Şekercioğlu ÇH. Increasing awareness of avian ecological function. Trend Ecol Evol. 2006; 21: 464–471. 16762448 10.1016/j.tree.2006.05.007

[65] Kellermann JL, Johnson MD, Stercho AM, Hackett SC. Ecological and economic services provided by birds on Jamaican blue mountain coffee farms. Conserv Biol. 2008; 22: 1177–1185. 10.1111/j.1523-1739.2008.00968.x 18616745

[66] Gove AD, Hylander K, Nemomissa S, Shimelis A, Enkossa W. Structurally complex farms support high avian functional diversity in tropical montane Ethiopia. J Tropic Ecol. 2013; 29: 87–97.

[67] Engelen D. Comparing avifauna communities and bird functional diversity of forest and farmland in southwest Ethiopia, M. Sc. Thesis, Stockholm University, Sweden; 2012.

[68] Gove AD, Hylander K, Nemomissa S, Shimelis A. Ethiopian coffee cultivation-Implications for bird conservation and environmental certification. Conserv Lett. 2008; 1: 208–216.

[69] Langellotto GA, Denno RF. Responses of invertebrate natural enemies to complex-structured habitats: a meta-analytical synthesis. Oecologia. 2004; 139: 1–10. 14872336 10.1007/s00442-004-1497-3

[70] Lichtenberg JS, Lichtenberg DA. Predation of caterpillars on understory saplings in an Ozark forest. Southeast Natur. 2003; 2: 423–432.

[71] Lemessa D, Hambäck P, Hylander K. The effect of local and landscape level land-use composition on predatory arthropods in a tropical agricultural landscape. Land Ecol. 2015; 30: 167–180.

[72] Nyffele M. Prey selection of spiders in the field. J Arachnol. 1999; 27: 317–324.

