# Supplementary material for: Arthropod but Not Bird Predation in Ethiopian Homegardens Is Higher in Tree-Poor than in Tree-Rich Landscapes
Source: PLoS One. 2015 May 11;10(5):e0126639. doi: 10.1371/journal.pone.0126639 (PMC4427475; doi:10.1371/journal.pone.0126639)
Supplement: S1 Tables — First step analyses with repeated measure using linear mixed effects model to test the effect of data collection time on predation rates of birds (Table A1) and arthropods (Table A2). The linear mixed effects model analysis on the effect of altitude on predation rates of birds (Table B1) and arthropods (Table B2). The linear mixed effects model analysis on the effect of plant type (coffee and avocado, on which plasticine caterpillars were attached) on predation rates of birds (Table C1) and arthropods (Table C2). The linear mixed effects model analysis to test the difference in predation rates between birds and arthropods on plasticine caterpillars (Table D) (PDF). (PDF) [file pone.0126639.s001.pdf]

S1 Tables. The linear mixed effects model analysis for the daily predation rates.

Table A. First step analyses with repeated measure using linear mixed effects model to test the effect of data collection times

1. Birds

```
> m1<-lmer(birdrateday~landtypes*gardentypes*jdat+ (1|sites)+(0+jdat|sites))
> Anova(m1)
Analysis of Deviance Table (Type II Wald chisquare tests)
```

Response: birdrateday			
	Chisq	Df	Pr(>Chisq)
landtypes	0.0089	1	0.9249
gardentypes	0.2819	1	0.5954
jdat	0.3878	1	0.5335
landtypes:gardentypes	0.3269	1	0.5675
landtypes:jdat	0.0182	1	0.8927
gardentypes:jdat	0.618	1	0.4318
landtypes:gardentypes:jdat	0.0025	1	0.9604

```
> m2<-lmer(birdrateday~landtypes*gardentypes+landtypes*jdat+gardentypes*jdat+(1|sites)+(0+jdat|sites))
> Anova(m2)
Analysis of Deviance Table (Type II Wald chisquare tests)
```

Response: birdrateday			
	Chisq	Df	Pr(>Chisq)
landtypes	0.0089	1	0.925
gardentypes	0.2823	1	0.5952
jdat	0.3876	1	0.5335
landtypes:gardentypes	0.3273	1	0.5672
landtypes:jdat	0.0182	1	0.8928
gardentypes:jdat	0.6187	1	0.4315

```
> m3<-lmer(birdrateday~landtypes*gardentypes+gardentypes*jdat+(1|sites)+(0+jdat|sites))
> Anova(m3)
Analysis of Deviance Table (Type II Wald chisquare tests)
```

Response: birdrateday			
	Chisq	Df	Pr(>Chisq)
landtypes	0.0099	1	0.9209
gardentypes	0.2813	1	0.5958
jdat	0.4859	1	0.4857
landtypes:gardentypes	0.3265	1	0.5677
gardentypes:jdat	0.6189	1	0.4315

```
> m4<-lmer(birdrateday~landtypes*gardentypes+jdat+(1|sites)+(0+jdat|sites))
> Anova(m4)
Analysis of Deviance Table (Type II Wald chisquare tests)
```

Response: birdrateday			
	Chisq	Df	Pr(>Chisq)
landtypes	0.0097	1	0.9217
gardentypes	0.2815	1	0.5957
jdat	0.4855	1	0.4859
landtypes:gardentypes	0.3373	1	0.5614

```
> m5<-lmer(birdrateday~landtypes+gardentypes+jdat+(1|sites)+(0+jdat|sites))
> Anova(m5)
Analysis of Deviance Table (Type II Wald chisquare tests)
```

Response: birdrateday			
	Chisq	Df	Pr(>Chisq)
landtypes	0.0097	1	0.9216
gardentypes	0.2817	1	0.5956
jdat	0.4886	1	0.4845

2. Arthropods

```
> fm1<-lmer(arthrateday~landtypes*gardentypes*jdat+ (1|sites)+(0+jdat|sites))
> Anova(fm1)
```

Analysis of Deviance Table (Type II Wald chisquare tests)

Response: arthrateday			
	Chisq	Df	Pr(>Chisq)
landtypes	1.5195	1	0.2177
gardentypes	0.0257	1	0.8726
jdat	0.0331	1	0.8556
landtypes:gardentypes	0.5085	1	0.4758
landtypes:jdat	0.7477	1	0.3872
gardentypes:jdat	0.7465	1	0.3876
landtypes:gardentypes:jdat	0.2060	1	0.6499

```
> fm2<-lmer(arthrateday~landtypes*gardentypes+landtypes*jdat+gardentypes*jdat+(1|sites)+(0+jdat|sites))
> Anova(fm2)
```

Analysis of Deviance Table (Type II Wald chisquare tests)

Response: arthrateday			
	Chisq	Df	Pr(>Chisq)
landtypes	1.5215	1	0.2174
gardentypes	0.0257	1	0.8725
jdat	0.0332	1	0.8555
landtypes:gardentypes	0.5089	1	0.4756
landtypes:jdat	0.7488	1	0.3869
gardentypes:jdat	0.7473	1	0.3873

```
> fm3<-lmer(arthrateday~landtypes*gardentypes+gardentypes*jdat+(1|sites)+(0+jdat|sites))
> Anova(fm3)
```

Analysis of Deviance Table (Type II Wald chisquare tests)

Response: arthrateday			
	Chisq	Df	Pr(>Chisq)
landtypes	1.5274	1	0.2165
gardentypes	0.0264	1	0.871
jdat	0.0351	1	0.8514
landtypes:gardentypes	0.5057	1	0.477
gardentypes:jdat	0.7505	1	0.3863

```
> fm4<-lmer(arthrateday~landtypes+gardentypes+jdat+(1|sites)+(0+jdat|sites))
> Anova(fm4)
```

Analysis of Deviance Table (Type II Wald chisquare tests)

Response: arthrateday			
	Chisq	Df	Pr(>Chisq)
landtypes	1.5269	1	0.2166
gardentypes	0.0264	1	0.871
jdat	0.0345	1	0.8527

Table B. Linear mixed effects model analysis on the effect of altitude on predation rates of birds and arthropods

1. Birds

```
> mod1<-lmer(bird_pred~landscapes+alt+(1|site))
 > Anova(mod1)
Analysis of Deviance Table (Type II Wald chisquare tests)
```

Response: bird_pred			
	Chisq	Df	Pr(>Chisq)
landscapes	0.0194	1	0.8893
alt	0.5958	1	0.4402

```
> mod2<-lmer(bird_pred~alt+(1|site))
 > Anova(mod2)
 Analysis of Deviance Table (Type II Wald chisquare tests)
```

Response: bird_pred			
	Chisq	Df	Pr(>Chisq)
alt	0.7942	1	0.3728

2. Arthropods

```
> model1<-lmer(arthro_pred~landscapes+alt+(1|site))
 > Anova(model1)
 Analysis of Deviance Table (Type II Wald chisquare tests)
```

Response: arthro_pred			
	Chisq	Df	Pr(>Chisq)
landscapes	11.6832	1	0.0006307***
alt	1.2067	1	0.2719812

Signif. codes: 0 ‘***’ 0.001 ‘**’ 0.01 ‘*’ 0.05 ‘.’ 0.1 ‘ ’ 1

Table C. The linear mixed effects model analysis on the effect of plant type (coffee and avocado, on which plasticine caterpillars were attached) on predation rates of birds and arthropods

1. Birds

```
> m1<-lmer(bird_pred~plant+(1|site))
 > Anova(m1)
 Analysis of Deviance Table (Type II Wald chisquare tests)
```

Response: bird_pred			
	Chisq	Df	Pr(>Chisq)
plant	0.3678	1	0.5442

2. Arthropods

```
> m2<-lmer(arthropod_pred~plant+(1|site))
 > Anova(m2)
 Analysis of Deviance Table (Type II Wald chisquare tests)
```

Response: arthromean			
	Chisq	Df	Pr(>Chisq)
plant	0.3972	1	0.5285

Table D. The linear mixed effects model analysis to test the difference in predation rates between birds and arthropods on plasticine caterpillars

```
> fm1<-lmer(predation~landscapes+predators+hgs_str+landscapes:predators+hgs_str:predators+(1|site))
 > Anova(fm1)
 Analysis of Deviance Table (Type II Wald chisquare tests)
```

Response: predation			
	Chisq	Df	Pr(>Chisq)
landscapes	1.8303	1	0.17609
predators	2.7619	1	0.09653 .
hgs_str	0.0311	1	0.86005
landscapes:predators	8.2257	1	0.00413**
predators:hgs_str	0.0258	1	0.8724
Signif. codes: 0 ‘***’ 0.001 ‘**’ 0.01 ‘*’ 0.05 ‘.’ 0.1 ‘ ’ 1			

```
> fm2<-lmer(predation~landscapes+predators+hgs_str+landscapes:predators+(1|site))
 > Anova(fm2)
 Analysis of Deviance Table (Type II Wald chisquare tests)
```

Response: predation			
	Chisq	Df	Pr(>Chisq)
landscapes	1.8303	1	0.176097
predators	2.8053	1	0.093955 .
hgs_str	0.0316	1	0.858964
landscapes:predators	8.3549	1	0.003847**
Signif. codes: 0 ‘***’ 0.001 ‘**’ 0.01 ‘*’ 0.05 ‘.’ 0.1 ‘ ’ 1			

```
> fm3<-lmer(predation~landscapes+predators+landscapes:predators+(1|site))
 > Anova(fm3)
 Analysis of Deviance Table (Type II Wald chisquare tests)
```

Response: predation			
	Chisq	Df	Pr(>Chisq)
landscapes	1.8303	1	0.176095
predators	2.8484	1	0.091467 .
landscapes:predators	8.4832	1	0.003584**
Signif. codes: 0 ‘***’ 0.001 ‘**’ 0.01 ‘*’ 0.05 ‘.’ 0.1 ‘ ’ 1			
